# Evaluation of image analysis tools for the measurement of cellular morphology

**DOI:** 10.3389/fcell.2025.1572212

**Published:** 2025-05-15

**Authors:** Matthew D. Bourn, Lauren F. Daly, Jim F. Huggett, Julian Braybrook, Jeanne F. Rivera

**Affiliations:** National Measurement Laboratory, LGC Ltd., Teddington, United Kingdom

**Keywords:** morphological cell analysis, critical quality attribute (CQA), metrology, traceability, standardisation

## Abstract

Morphological cell analysis offers a means of identification and classification of key morphological measurement parameters linked to cell bioactivity and cell health and, as such, it is of great interest to academic and industrial research sectors. Widespread adoption of this approach has yet to occur, partially due to the lack of alignment in analysis methodologies and output metrics, limiting data comparability. Work within the cell metrology and wider multidisciplinary community aims to reduce data variability through the improved alignment of image acquisition and analysis methodologies. Furthermore, to improve data comparability, research has also focused on the identification of a minimal set of morphological measurands, often termed critical quality attributes (CQAs), which are traceable to standardised (SI) units of measurement. Whilst efforts in defining CQAs have progressed significantly for healthcare applications, there are still numerous measurement challenges associated with image analysis of cultured cells due, in part, to their complex heterogenous nature. This review evaluates the various automated image analysis tools developed for morphological analysis of four commonly considered cell morphological features: the nucleus, actin cytoskeleton, mitochondria, and the cell membrane. The measurement methodologies and outputs from each tool have been evaluated and coinciding outputs have been highlighted as potential CQAs.

## 1 Introduction

Morphological cell analysis utilises the data from cell microscopy images to generate quantitative data which portrays key information about the cells structure and cell bioprocesses (Chandrasekaran et al., 2021; Vincent et al., 2022; Stirling et al., 2021). This practice, often referred to as cell profiling, involves the analysis of key morphological features of differing cell populations and organelles, which generally consists of analysing fluorescent intensity, shape features, and co-localisation of signals (Piccinini et al., 2017). Cell profiling is often used for high-throughput investigations during drug development to identify mechanisms of drug uptake and toxicity, by comparing cell morphologies of treated cells with those of untreated cells (Kang et al., 2016; Ziegler et al., 2021; Scheeder et al., 2018). Furthermore, advances in imaging technologies allow for analysis of complex cell models, such as organoids, for disease characterisation, and optimisation of immunotherapies - bridging the gap between preclinical and clinical research (Silva-Pedrosa et al., 2023). The consideration of cell morphology overcomes limitations of alternative analysis methods such as plate-based viability assays or cytometry-based expression assays, which are unable to discriminate subtle morphological variations (Chen et al., 2016). Although morphological cell analysis is a desirable technique which provides extensive information about the state of various cell populations, there are still numerous challenges within the workflow of cell profiling that need to be addressed to facilitate its adoption by the wider scientific community. A particular application for image-based cell profiling is during drug development process, due to its high-throughput potential to assess large datasets and improve analysis efficiency. However, discrepancies on the quality of training data sets influence the unbiased strategy of image-based profiling. The definition of good quality data for training sets remains unclear, though some QC guidelines for tools like Cell Painting assays for well-characterized cell lines (e.g., A549) and data preprocessing methods in CellProfiler enhance image-based profiles (Garcia-Fossa et al., 2023; Assefa et al., 2023). Furthermore, continuous improvements in experimental workflows are also increasingly being published, that should address challenges associated with morphological perturbations (Cimini et al., 2023).

Cellular analysis through evaluation of morphology derived from bio-imaging techniques is an important emerging area of cell metrology (Faruqui et al., 2020). Metrology is the science of measurement and its application to cell analysis (cell metrology) aims to improve the accuracy of methods for cell measurement. Central to cell-based measurement is an accurate definition of the identity of the cells being analysed. This also represents an expanding area of science as cell measurements advance. For example, the identification of a set of morphological attributes, often referred to as critical quality attributes (CQAs), associated with cellular bioactivity is key to advancing morphological cell analysis (Lin-Gibson et al., 2016). Furthermore, the establishment of material standards to allow metrological traceability would greatly improve the reproducibility of measurements.

Whilst identification of CQAs would aid cell measurements, the lack of workflow standardisation relating to cell organelle staining, image acquisition, analysis tools, and mathematical analysis models currently contributes to undetermined variations in morphological measurement data. Efforts to remove and reduce these challenges have been made throughout the field of digital pathology where the analysis methods of images derived from histological samples have been developed significantly to enable precise characterisation and improve clinical diagnostic practices. Furthermore, ISO (International Organisation of Standardisation) standards related to the use of AI and image analysis are currently under development (ISO/AWI 24051–2 Part 2: Digital pathology and artificial intelligence-based image analysis under ISO/TC212 (Bankhead, 2022; ISO, 2025a). However, these analytical methods cannot be applied to cell culture-based images due to their morphological complexity and heterogeneous nature. Documentary standards such as ‘ISO/CD23511: Biotechnology-General requirements and considerations for cell line authentication under ISO/TC276’ are beginning to identify and address the challenges in this field (ISO, 2025b). These efforts highlight the critical need for precise methodologies to improve measurement confidence, support global efforts in biotechnology, and enable increased translation of research evidence into practice.

Development of a set of robust, validated methods and a concise set of high-confidence measurement parameters is reported to be pivotal for the functionality of morphological cell profiling and would further facilitate its utilisation throughout the research and biomanufacturing sectors (Plant et al., 2014; Plant et al., 2018; Faruqui et al., 2020). The ability to accurately measure viable cell count, classify the number of proliferating, senescent and quiescent cells, and to identify apoptotic cells using morphological cell analysis offers a means of improving quality control in cell therapeutic product manufacturing, viral vector manufacturing, and protein production (Lin-Gibson et al., 2016; Simon et al., 2016; Sarkar et al., 2017; Huang et al., 2021; Pierce et al., 2021). Morphological cell profiling could provide complementary data to existing biological techniques such as molecular profiling, protein biomarker detection and plate-based assays and provide a link across multiple analysis methodologies. For example, morphological profiling of mesenchymal stem cells (MSCs) may provide a means of characterising their functional heterogeneity and quantifying their therapeutic potency (Phinney, 2012). In another example, Treiser et al. have previously demonstrated the ability of actin cytoskeleton morphological analysis to predict the fate of MSCs grown on differing substrates (Treiser et al., 2010). ISO have established a dedicated committee (Biotechnology ISO/TC 276/SC1) that primarily aims to publish standard documents outlining general guidance and key considerations for cell counting methodologies and evaluation of CQAs. These include, but not limited to, ‘the determination of the intended use of the cells’ and ‘awareness of the cell morphology under the microscope’ and are currently working towards further guidance for cellular morphological analysis (Huang et al., 2021; Allocca et al., 2024).

The Cells Analysis Working Group (CAWG), under the Consultative Committee for Amount of Substance (CCQM) metrological organisation, is also working towards improving the global comparability of cell-based measurements and identification of CQAs by performing interlaboratory comparison studies across international metrological research institutes (CCQM, 2021). Together, these organisations are facilitating measurement comparability of morphological cell profiling. The benefits of measurement standardisation can be observed in other cell-based techniques such as flow cytometry, where efforts from international comparative studies resulted in the development of candidate reference materials for CD4^+^ cell counting for HIV/AIDS diagnosis. These studies documented measurement uncertainty, a critical biomarker for these cell type of interest (WHO BS/10.2153) and proficiency testing through providers such as the National External Quality Assessment Scheme (NEQAS). NEQAS aims to improve global diagnostic testing for improved quality of care by providing flow cytometry programmes (Stebbings et al., 2015; Rajagopal et al., 2020; NEQASUK, 2024). Similar types of material standards may offer an additional route needed to guide morphological cell analysis.

Since cell imaging focuses on morphological features such as cell nuclei, actin cytoskeletons, mitochondria networks and cell membranes, these will be the primary cell components considered throughout this review. The method by which images are analysed likely exhibits the greatest amount of variation across this field, and multiple tools and analysis packages have been developed to accommodate a range of purposes. This review will highlight cell staining and imaging strategies focusing primarily on compiling and assessing the various tools developed for the analysis of each cellular component and adjacent bioprocesses. The metric outputs from each analytical tool will be discussed with reference to the identification of a minimal set of CQAs that encompass key measurements related to cell bioactivity. Reducing the number of potentially redundant measurands would streamline cell characterisation and link measurements directly to the cell products intended use. Thus, a set of CQAs may consist of singular measurements of cell structures along with measurements of the relationship between structures, to fully describe the confirmational changes occurring within each cell. This review will discuss potential CQAs in reference to their ability to be expressed in standardised units (SI) of measurement. This is an essential characteristic for any potential CQA as it enables traceability back to a reference material, therefore facilitating data comparability across multiple instruments and laboratories. Such metrological considerations are often overlooked when developing analysis tools and methodologies, which can hinder adoption across the wider scientific community.

## 2 Cell imaging strategies

### 2.1 Imaging technologies

Several types of fluorescence microscopes can be used for morphological profiling with confocal fluorescent microscopy being one of the most utilised. Confocal microscopes are widely available and possess the ability to produce detailed three-dimensional (3D) Z-stacks of cells across multiple fluorescent channels. The primary drawbacks of capturing Z-stacks involve the slow acquisition times and phototoxicity effects when imaging live cells (Ojha and Ojha, 2021; Douthwright and Sluder, 2017). Live cell imaging allows for continuous observation of cell morphologies as the bioprocesses occur, providing that temperature and CO_2_ conditions are maintained. However, limitations associated with phototoxicity and stain incompatibility with live cells often renders live cell imaging unfeasible (Purschke et al., 2010). Cell fixation can instead be used to preserve cells in a life-like state, allowing for a snapshot of cellular morphology to be acquired. Fixation is advantageous for many antibody-based assays, as, when combined with permeabilisation, allows for access to intracellular structures (Fischer et al., 2008).

Widefield fluorescent microscopy allows for faster image acquisition at the cost of reduced image detail from 2D images. Profiling of large numbers of cells within a population is a key requirement for many morphological analysis studies, such as high content screening, therefore acquisition time is a key concern when choosing a methodology. Spinning disc confocal microscopy (SDCM) offers an alternative to conventional confocal microscopy, due to its ability to overcome the time constraints of Z-stack acquisition (McKayed and Simpson, 2013). The widespread adoption of SDCM is evident by the increasing number of publications and applications using this type of microscopy leading to standardisation efforts in quality assessment in light microscopy (Faklaris et al., 2022; Ahmadian et al., 2024; Poole and Mostaço-guidolin 2021). Despite this, laser scanning confocal microscopes currently remain more commonly used particularly throughout the research community (Patil-Takbhate et al., 2024; McKayed and Simpson, 2013).

The choice of imaging system is also dependent on the analytical tool being used for image analysis. Complex analysis tools that investigate the intricate networks formed by actin cytoskeletons or mitochondria, often require 3D images with finely sliced Z-stacks to accurately determine linkages in the Z-axis (Harwig et al., 2018; Sahu et al., 2024). The minimum magnification required to produce a sufficiently detailed image for morphological cell analysis is dependent on the feature of interest, staining quality, image gain, and background noise levels. The processing of images post-acquisition also introduces downstream morphological measurement uncertainties. Deconvolution, the process of improving the contrast and resolution of images using mathematical algorithms, is commonly used to remove out of focus light present in fluorescent images due to point spread function (PSF) effects (Wallace et al., 2025). Deconvolution tools are often incorporated into microscopy acquisition software, as intrinsic optical properties of the hardware, such as the numerical aperture, refractive indices, and quality of optical components are known. Several types of standalone, open-source deconvolution tools are also available such as DeconvolutionLab2 and Deconwolf (Wernersson et al., 2024; Sage et al., 2017). A range of algorithms can be implemented to perform image deconvolution, and can broadly be categorized into deblurring algorithms, which aim to remove out of focus light from images, and restorative algorithms which aim to reassign out of focus light in 3D images to the appropriate in-focus location in the Z-axis (Wallace et al., 2025). This tool is particularly useful for co-localisation studies where chromatic aberrations from differing fluorophores can lead to misalignment between acquisition channels (Mascalchi and Cordelieres, 2019). From a metrological perspective, the impact of these operations on the intensity and shape features determined from morphological cell analysis is yet to be fully investigated. Overall, this range of cell imaging strategies introduces a high degree of data variability, which presents further challenges in the alignment of cell morphological analysis methodologies.

Whilst some guidelines have been produced in ISO 21073:2019 that details standards on performance of microscopes for biological imaging and address challenges in reproducibility, further work is still required to address the additional challenges outside the scope of the guideline (ISO, 2019). This is further compounded by the lack of universally adopted microscope quality controls and instrument characterisation, when compared to other instrumentation such as flow cytometry, where reference standards and best practice guidelines now exist (DeRose et al., 2008; Cossarizza et al., 2021). Laser illumination power, field uniformity, axial resolution, and chromatic aberration have all been identified as key factors which must be characterised to facilitate the production of reproducible, quantifiable fluorescent image data (Montero Llopis et al., 2021). To address this need, the Quality Assessment and Reproducibility for Instruments and Images in Light Microscopy (QUAREP-LiMi) working group was formed and a set of ISO standards published describing the best practices for acquiring these measurands (Nelson et al., 2021; Hammer et al., 2021). It is recommended that a comprehensive set of image metadata should accompany published images and associated data, using what is known as the Open Microscopy Environment (OME) model (Hammer et al., 2021; Goldberg et al., 2005). Community-developed guidelines have also been published which aid authors in increasing the clarity and reproducibility of image figures (Schmied et al., 2023). Whilst this will undoubtedly improve the dependability and reproducibility of image quantification, many labs and users, specifically in academia, may be hesitant to adopt such exhaustive quality controls, due to time constraints and the advanced training required.

### 2.2 Morphological features as CQAs

Morphological profiling refers to the critical analysis of cells to identify quantifiable phenotypic metrics such as size, shape, intensity distributions and subcellular components. These features can be indicative of key bioprocesses such as apoptosis, proliferation, senescence and quiescence, that underpin cell health and function. Moreover, features such as blebbing, nuclear fragmentation and cell shrinkage can be utilised to enumerate cells undergoing these specific processes within a population (Ziegler and Groscurth, 2004; Yang et al., 2022). Identification of candidate CQAs is paramount to accelerate the translation of cell therapies. Whilst cell morphology demonstrates a potential candidate CQA, selecting a specific morphological feature can be challenging due to the measurement uncertainties associated with each cell characteristic. Furthermore, variation in image capture settings and analysis, due to several key methodology steps: cell type, staining method, imaging strategy, analysis method and output parameters, can also present further measurement challenges for biotechnology. A summary of the commonly used stains and reagents used for the visualisation of the nucleus, cell membrane, actin cytoskeleton, and mitochondria is given in Table 1.

**TABLE 1:** Summary table of common stains for each cell organelle/structure described throughout this review. Stain target/mechanism of action is described alongside notable references employing the use of each stain.

Organelle/ Structure	Stain	Target	References
Nucleus	DAPI	DNA-binding stain - Adenine-thymine rich regions of DNA	Janssen, Breusegem, and Larrieu, 2022; Faklaris et al., 2022; Bonte et al., 2023
	Hoechst 33342	DNA binding stain - Minor groove of ds-DNA with preference to AT rich regions	Janssen, Breisegem, and Larrieu, 2022; Okkelman et al., 2020; Mcquin et al., 2018
	NucSpot; Nuclear-ID	DNA binding stain	Oakley et al., 2021; Katiyar et al., 2022; Bissenova et al., 2023
	SYTOX	Nuclei and chromosomes	Thakur, Cattoni, and Nollmann, 2015; Demuynck et al., 2020
	Lamin A/C; Lamin B	Binds to the proteins found in the nuclear lamina	Gauthier and Valentine (2021)
	Y-H2AX; RAD51	Binds to damaged DNA producing nuclear foci	Nair et al., 2021; Liu et al., 2023; Hoppe et al., 2021; Rothkamm et al., 2010
	7-Aminoactinomycin D (7-AAD)	Intercalates within G-C rich regions of DNA in cells with compromised membranes	Shenkin, Baby, and Maiese, (2007)
Cell Membrane	Wheat Germ Agglutin	Glycans - binds to N-acetylglucosamine and N-acetylneuraminic acid (sialic acid) residues	Manning et al., 2017; Mcquin et al., 2018; Bray et al., 2017; Schoen et al., 2017a
	Concanavalin A	Glycans - α-mannopyranosyl and α-glucopyranosyl	Manning et al., 2017; Morgan et al., 2013
	CellTracker™	Compound freely passes into cells where it binds with amine and thiol groups in the cell membrane	Correa de Sampaio et al. (2012)
	CellBrite™	Based on lipophilic carbocyanine dyes	Shannon et al., 2024; Zhang et al., 2018
	PKH	Lipid Biolayer – Incorporated aliphatic reporter molecules into the cell membrane lipid biolayer by selective partitioning	Shimomura et al., 2021; Takahashi et al., 2020
	CellMask™	Areamphipathic molecules which are lipophilic	Liu et al., 2024; Mcquin et al., 2018; Tamima et al., 2023
Actin	Phalloidin	Selectively labels F-actin within the cell	Pospich, Merino, and Raunser, 2020; Ziegler et al., 2021; Mazloom-Farsibaf et al., 2021; Kumari et al., 2020; Mishra et al., 2019
	LifeACT™	Selectively labels F-actin within the cell	Mazloom-Farsibaf et al., 2021; Kumari et al., 2020
	SiR-Actin/XSiR-Actin	Selectively labels F-actin within the cell	Pospich, Merino, and Raunser, 2020; Mishra et al., 2019; Nasufovic et al., 2025
Mitochondria	Mitotracker™	Rosamine-based or Carbocyanine stain. Accumulates in active mitochondria	Samanta et al., 2019; Desai et al., 2024
	TMRM	Cationic dye that accumulates in healthy mitochondria	Samanta et al., 2019; Desai et al., 2024; Okkelman, Papkovsky, and Dmitriev, 2020
	JC-1	Cationic carbocyanine dye that accumulates in mitochondria	Okkelman, Papkovsky, and Dmitriev, 2020; Sivandzade, Bhalerae, and Cucullo, 2019

#### 2.2.1 Nuclear morphology

The nucleus is one of the most commonly studied cellular organelles, with the responsibility of compartmentalising the cell’s genetic material and regulation of gene expression. The morphology of the nucleus can vary between cell types and can change depending on the overall health of the cell. The nuclear structure is made up of the nuclear envelope (NE) and the nuclear matrix. The NE is a double membrane, which regulates the transportation of molecules from in and out of the nucleus (Fischer, 2020). The most common aberrations of the nucleus include abnormal sizing, blebbing, and NE invaginations which all indicate signs of poor cell health and the development of disease (Fischer, 2020; Schoen et al., 2017a; Gauthier and Valentine, 2021; Janssen et al., 2022). As well as nuclear morphology being symptomatic of disease, their morphology can also be indicative of current cell cycle status. For instance, cells undergoing mitosis lose their nuclear envelope and exhibit nuclear fragmentation whereas, cells undergoing cell cycle arrest present a flattened nucleus with increased surface area (Ziegler and Groscurth, 2004; Smoyer and Jaspersen, 2014; Pathak et al., 2021).

As nuclear morphology can be indicative of cellular health and specific biological processes, it is an ideal candidate for cellular profiling and is a commonly considered organelle for cell morphological analysis. A range of nuclear and DNA stains and antibodies are available for visualisation of the nucleus such as Hoechst 33342, DAPI, NucSpot, and Lamin A/C. Many stains are cell impermeable, allowing for live/dead cell discrimination as only cells with a compromised nuclear membrane show fluorescence.

#### 2.2.2 Cell membrane

The structure of a cell membrane can provide a wealth of information on the bioprocesses occurring within a cell (Denz et al., 2017; Zhukov and Popov, 2023). The membrane provides information on the overall size and surface area of the cells and can be used to classify cells in various cellular processes. For example, the area of the cell can be utilised to classify cells undergoing proliferation due to an increase in size and apoptosis due to cellular shrinkage (Zhang et al., 2018). The analysis of cell membrane morphologies also allows for the characterisation of subtle morphological changes visible using microscopy, which are often unable to be identified when using analysis methods such as flow cytometry and plate-based assays. The complete staining of membranes also facilitates cell segmentation in confluent cell monolayers as cell membrane staining is often more intense at the periphery. This can be a valuable tool when aiming to separate individual cell cytoskeletons or mitochondrial networks, which can provide further information on the bioprocesses occurring.

Analysis of cell membrane morphologies can provide a range of quantitative information depending on the method by which membranes are stained. In addition to morphology, several membrane impermeant stains, such as Propidium Iodide and 7-AAD, which allow for the membrane integrity of the cell to be probed. In general, these stains function by fluorescing only when bound to DNA inside the cell, thus identifying non-viable cells with compromised membranes. These staining strategies provide quantifiable mean intensity readouts and, when combined with morphological-focused stains, allow for complete morphological profiles of cells to be established.

#### 2.2.3 Actin cytoskeleton

The actin cytoskeleton provides internal structure to a cell and participates in various cellular processes including mitosis, migration, contraction and elongation, and adhesion (Povea-Cabello et al., 2017; Gosak et al., 2022). The two forms of actin: filamentous (f-) and globular (g-) actin, exist in a state of flux that is regulated by actin binding proteins (Kumari et al., 2020). The extent and structure of the actin filament polymerisation within a cell is dependent on the bioprocesses occurring. During cytokinesis, the final stage of mitosis, actin cytoskeletons from a contractile ring, which contracts to cleave the cell in two. During apoptosis, caspase activation can also induce cells to show similar contractile ring structures as part of programmed cell death (Ren et al., 2021). Actin plays a crucial role in each of these distinct processes which result in comparable morphological change. This highlights the potential of morphological cell profiling to identify cell bioprocesses, however, a complete set of morphological data is required to accurately do so (Zhang et al., 2023). Characterisation of the cytoskeletal morphology can inform on cell status and provide a useful tool for cell profiling (Leemreis et al., 2006). In addition to morphological profiling, several alternative methods of determining cytoskeletal function exist in the form of techniques which look to probe cell stiffness using deformation. Optical tweezers, atomic force microscopy, and microfluidic deformation have all previously been used to probe cell membrane stiffness and infer the functionality of the actin cytoskeleton (Jokhadar et al., 2021; Daniel et al., 2023; Su et al., 2022). Whilst these techniques are informative, many suffer from low throughput cell analysis and are not easily multiplexed with additional analysis targets, such as other cell organelles, and do not allow for direct observation of the actin cytoskeleton morphology. Nevertheless, the quantifiable output from these analysis methods can serve as a means of validating actin morphology observations and aid in the identification of morphological CQAs associated with actin functionality.

Actin and cell membrane profiling generate many overlapping outputs related to cell status which can aid in the development of multiparameter outputs for each of the cellular processes mentioned above. Multiparameter outputs increase cell profiling robustness and reduce the impact of staining artefacts on cell characterisation. Analysis of mammalian cytoskeletons will be the primary focus here; however, it should be noted that other sophisticated analysis tools have been developed with the aim of analysing plant actin cytoskeletons due to their complex morphologies. For the visualisation of actin, Phalloidin is typically regarded as the gold standard in actin staining. However, well characterised compounds such as SiR-actin, Lifeact peptide and recently developed SiR-XActin offer alternative fluorescent probes that overcome the fixation limitations of phalloidin for live cell imaging (Nasufovic et al., 2025; Takagi et al., 2021; Mazloom-Farsibaf et al., 2021). This has facilitated the real-time imaging of actin cytoskeleton and visualisation of confirmational changes using live cell, time-lapse imaging.

#### 2.2.4 Mitochondria

Mitochondria are maternally inherited membrane-bound organelles conserved within all eukaryotic cells. Their main function is to perform aerobic respiration and provide the cell with chemical energy in the form of ATP. As such, mitochondria are responsible for the homeostatic maintenance of many cellular processes including programmed cell death, mitosis, reactive oxygen species production and calcium signalling (Brand et al., 2016). Due to their dynamic roles, the morphology and quantity of mitochondria can vary within the cell based on the cell’s viability, function, and energy requirements–presenting an ideal target for cell profiling. Structurally, the mitochondria are defined by their compartmentalised structure consisting of an inner and outer membrane that shift from small, fragmented units to larger, elongated, interconnected networks. The assembly and disassembly of the intracellular networks are controlled and regulated through two opposing processes, fission (disassembly), and fusion (assembly). The morphology of the mitochondrial networks is directly related to the energy demands within the cell, and in turn, be used as an indicator of specific cellular processes. (Herrera et al., 2018; Otera and Mihara, 2012).

Due to the importance of mitochondrial morphology in characterising and studying cell health and biogenesis, high quality imaging is required for successful phenotyping. Several different mitochondrial stains can be utilised depending on the morphological information required for the study. Quantification of mitochondrial morphology has proven to be challenging, and early efforts were based on unreliable and inconsistent qualitative descriptors. For example, mitochondrial morphologies are commonly classified as elongated, fragmented, or collapsed and their heterogeneity within the cell were not taken into consideration. This may lead to subjective results and inconsistencies with inter-laboratory comparison studies. To coincide with the quantification of mitochondrial morphology CQAs, mitochondrial functionality in relation to cell health can be assessed through several plate-based assays. These assays can measure; ATP, reactive oxygen species (ROS), cell oxygen consumption, and Ca^2+^ content (Yin and Shen, 2022). Whilst these assays allow for a determination of mitochondrial activity, they are typically end-point assays which are not easily multiplexed with additional measurements such as morphology. Furthermore, on their own, they may not reflect accurately with cell viability and proliferative status due to mitochondrial hyperactivity (Rai et al., 2018).

## 3 Analytical tools for morphological analysis

Numerous open-source image analysis software tools are available for cell-based image analysis, with differing capabilities and limits. Examples include Icy, CellProfiler, ILASTIK, and ImageJ with a host of user-developed tools for the analysis of specific cellular processes, such as mitochondrial fission/fusion and actin cytoskeletal rearrangement (Wiesmann et al., 2015). In addition to the variability introduced by numerous software sources, morphology can be unique to a particular cell type which creates further difficulties in analysis. Bray et al. aimed at addressing these issues by developing a ‘Cell Painting’ assay (Bray, Singh, et al., 2017); which outlined detailed methodologies for staining, imaging and analysis of cell morphologies using CellProfiler (Singh et al., 2014; Bray et al., 2017; Mcquin et al., 2018; Stirling et al., 2021). This assay marked a step towards the standardisation of cell morphological assessment, however challenges related to image acquisition and microscope standardisation still remain. Furthermore, whilst versatile, CellProfiler is limited in the measurement and quantification of specific organelle morphologies. This section will highlight analytical tools developed for the measurement of specific organelles and evaluate their outputs in relation to CQAs for each organelle. As it will be seen, the range of applications and analysis tools available for morphological cell profiling presents challenges in the determination of concise morphological parameters, which are comparable and traceable to a standard set of measurement values (Marklein et al., 2018).

### 3.1 Nuclei image analysis methodologies and tools

A broad range of automated image analysis tools and methods are available to researchers performing morphological cell analysis. A key feature of any nucleic analysis tool, is the accurate detection and segmentation of individual cell nuclei. Nuclei are frequently used as initial seed points for the detection of associated cell organelles in multi-parameter fluorescent images, meaning nuclei segmentation inaccuracies can propagate significant uncertainties throughout the analysis pipeline. Several factors such as cell type, confluency, stain homogeneity, and imaging methodology all influence the complexity of effective nuclei segmentation and present measurement challenges that need to be addressed. This is further complicated in particular cell types that commonly display polynucleation, such as HeLa cells, which has led to over-segmentation of individual cells. Furthermore, in densely populated cultures where cells often overlap, simplistic methods of analysis are often insufficient in accurately identifying individual nuclei. Several computational methodologies have been developed to address this issue, which employ the use of graph-cut, convex-concave, and contour analysis to segment overlapping or closely associated nuclei. These methodologies have been found to produce results which align more closely to manually identified nuclei counts (Zhou et al., 2019; Yi et al., 2019; Qi, 2014). Recent advances of incorporating deep learning algorithms into analytical tools have further enabled the automation of image analysis (Hollandi et al., 2022). This reduction in user input aids in the reduction of user analysis bias and improves interlaboratory comparability. However, the choice of input parameters and mathematical models used can impact AI-driven analysis, and further work is needed before fully unsupervised analysis can be fully relied upon.

Tools range in their complexity, from simplistic tools which require significant user input and quality control, to sophisticated tools which employ the use of machine-learning and training datasets. CellProfiler, a commonly used tool, employs minimum cross-entropy or OTSU thresholding methodologies combined with local maxima declumping to segment nuclei. These methods are effective for sparse cells with uniformly stained nuclei, however the declumping algorithms are limited when nuclei become closely associated in confluent cell samples. This inhibits the development of standardised pipelines due to the requirement for user quality assurance. Ilastik, an interactive machine-learning based tool, bridges the gap between pure user defined, and pure AI-driven analysis tools by allowing the user to define pixel classes and input examples of each class (Berg et al., 2019). Ilastik then assigns each pixel to a class using a Random Forest classifier. However, since pixel classification is computed from a spherical neighbourhood of pixels, object level shape characteristics are not considered, preventing segmentation of closely associated nuclei. Users are able to input segmentations obtained outside of Ilastik to overcome this limitation. Cellpose 2.0 presents a tool in which users can use either pre-trained or user-trained models to achieve cell and nuclei segmentation (Berg et al., 2019). A neural network is used to segment images based off as little as 100-200 user defined regions of interest, a significant reduction in the number of features generally required for the training of such complex models. The application of pre-trained models presents a potential means of standardising segmentation methodologies across multiple laboratories, provided that prior staining and imaging parameters remain constant. Ershov et al. have also developed an automated tracking software, TrackMate 7, which incorporates popular segmentation and analysis tools (MorphoLibJ, Weka, Ilastik, StarDist, and CellPose) into a complete FIJI plugin. This allows users to use complex segmentation and analysis approaches in a tool which is freely available and able to be continuously developed by users (Arganda-Carreras et al., 2017; Stevens et al., 2022; Legland et al., 2016; Jacquemet et al., 2020).

Typically, measurements outputs from nucleic analysis tools include shape (area, diameter, perimeter, and eccentricity) and intensity (mean, edge, integrated, and granularity). Measurements of shape are generally traceable to the S.I unit of meter, aside from circularity values, whereas intensity metrics are primarily expressed in arbitrary units which require comparison with reference material intensity values to produce standardised measurements. Principle component analysis (PCA) can be used to determine the relationship between each measurement to reduce the number of necessary metrics to a minimum essential set. These metrics are often sufficient for many purposes, such as the identification of condensed DNA in mitotic cells and fragmented DNA in apoptosis (Jevtić et al., 2014; Janssen et al., 2022). Simplistic shape and intensity measurements can, however, often overlook subtle variations in nucleic morphology which may allude to variations in bioactivity. As a result, analysis, and quantification of NE morphology has been of particular interest in morphological cell profiling (Schoen et al., 2017a). Janssen et al. has reviewed in further detail the current quantification methods for several nuclear abnormalities (Janssen et al., 2022b).

**FIGURE 1:**
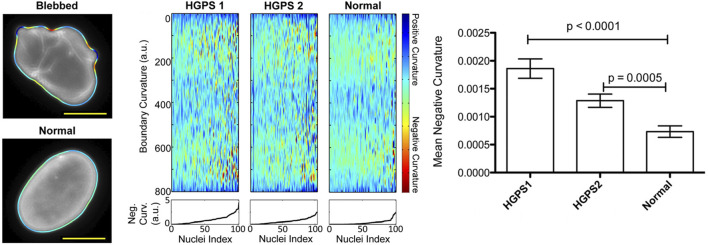
Overview of Driscoll’s mean negative curvature analysis showing identification of nuclei curvature for a blebbed, HGPS nucleus and normal, oval-shaped nucleus. Plots of boundary curvature are shown as heat maps with each vertical line corresponding to a single nuclear measurement. Mean negative curvatures for each type of nucleus are shown in the bar chart (Driscoll et al., 2012). Figures have been adapted from publications with permissions.

To overcome challenges associated with variations in nucleic morphology, Phillip et al. have developed the Visually Aided Morpho-Phenotyping Image Recognition (VAMPIRE) software tool for the quantification of cell nuclei and membrane morphologies (Phillip et al., 2021). This tool uses unsupervised, machine-learning based analysis and classification based on the fitting of equidistant points along cell and nuclear contours. This program demonstrated the potential of machine-learning to accurately identify, quantify, and classify nuclear shape through the use of contours. However, this quantification approach revealed that metrics such as solidity, aspect ratio, and shape factor are often insufficient in identifying morphological heterogeneity in cell nuclei and membranes. Moreover, VAMPIRE is unable to segment individual cells in an image, so it is reliant on additional analysis tools to perform this pre-processing. The accuracy of the data output from VAMPIRE is therefore heavily reliant on the effectiveness of the segmentation tool used and presents additional measurement uncertainty (Phillip et al., 2021). Driscoll et al. also developed a custom MATLAB code which analysed the mean negative curvature (MNC) of the nuclear shape using an active contour-based boundary extraction algorithm. This tool allows identification of irregular nuclei shapes caused by a disease which induced mutant Lamin A scaffolding and exhibit and outlines of blebbed and normal nuclei alongside measurement outputs of mean negative curvature analysis (Figure 1) (Driscoll et al., 2012). As with VAMPIRE, this approach presented a further limitation on impact variations in pixel size and smoothing have on the calculation of MNC. This presents additional challenges when looking to perform an interlaboratory comparison with multiple microscopes, highlighting the need for the standardisation of imaging methodologies.

Automated image analysis tools have also been developed to quantify the degree of DNA damage within a nucleus. McDonough et al. developed a tool, which segments and classifies cell nuclei based on DNA fluorescence imaging with DNA damage-stained cells used as a training dataset (Mcdonough and Vadivazhagu, 2020). Outputs of this tool were separated into shape-based and texture-based features to determine the accuracy of each set of these features in the classification of cells with DNA damage. Haralick features are textural measurements based on grey-level intensities of pixels of an image, such as contrast, sum variance, difference variance, correlation, and entropy were all found to score highly as features which are capable of distinguishing between cellular classes (Haralick et al., 1979; Candelero et al., 2020). These features present metrics which may be considered as potential CQAs which could accompany shape-based features such as nuclei area and curvature measurements. This analysis technique, as a quantitative method for characterisation of 3D dosimetric distributions derived from clinical samples, demonstrates a potential application of texture-based image analysis. Overall, it is apparent that nuclear morphology can be analysed and quantified using a range of differing analysis tools which produce output metrics which vary in their complexity. The standardisation of measurement methodologies and reported values will be achieved through coordination between academic, industrial, and metrological research sectors.

### 3.2 Cell membrane image analysis methodologies and tools

**FIGURE 2:**
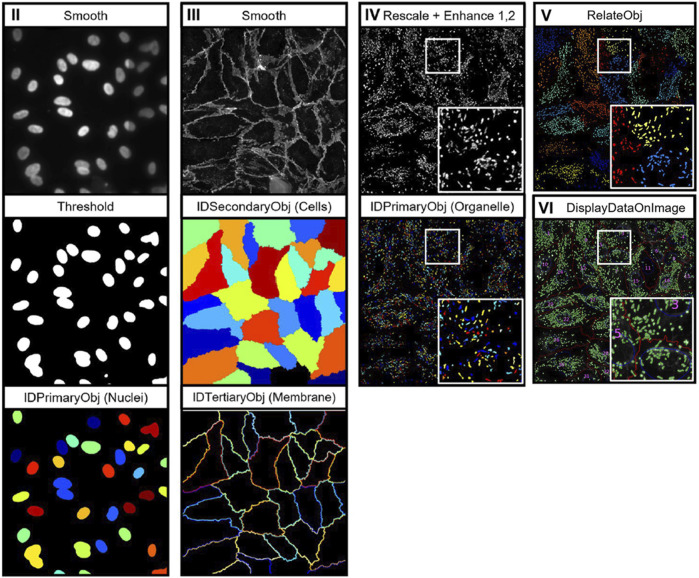
Depiction of the image analysis workflow for OrganelleProfiler. Individual organelle images are smoothed and thresholded, then primary (nuclei), secondary (cells), and tertiary (organelle) objects are identified using the associated CellProfiler modules. Tertiary objects are then related to individual cells as shown in image pane (V) (Laan et al., 2023). Images reproduced from publications with permissions.

In comparison with other cell structures, cell membrane morphological analysis can largely be performed without the use of specialised analysis tools. However, as with nuclei analysis, segmentation of cell membrane boundaries is key to achieving precise measurements of membrane morphology. Aforementioned tools, such as Cellpose 2.0, may also be required to segment cell membranes in densely populated images. Output metrics from cell membrane analysis consist of properties such as area, perimeter, and circularity, intensity, and edge intensity values (Mcquin et al., 2018; Schindelin et al., 2009; Berg et al., 2019). Laan et al. recently developed a CellProfiler-based pipeline for the analysis of cell nuclei, membrane, and organelles, known as OrganelleProfiler (Laan et al., 2023). Figure 2 depicts the image analysis workflow which uses tiered identification to identify primary (nuclei), secondary (cell), and tertiary (organelle) objects. Analysis of endothelial colony forming cells (ECFCs) exposed to cytoskeletal drugs demonstrated the pipelines’ ability to quantify variations in cell morphology through the analysis of multiple cell organelles. However, as described previously, CellProfiler is user-dependent and often requires manual inspection of output images to determine pipeline performance.

Specialised membrane morphological analysis tools such as Automated Cell Morphology Extractor (ACME) and MorphoGraphX have been developed for the reconstruction of cell membrane images taken of larger tissue structures (Mosaliganti et al., 2012; Reuille et al., 2015). ACME was shown to accurately segment and reconstruct cell membrane shapes from both 2D and 3D dense tissue images. The analysis algorithms used in ACME were inspired by those used in MRI and CT imaging to detect blood vessels, implementing Hessian-based filters to identify eigenvectors associated with cell boundaries. Cell size and shape metrics output from this tool demonstrated improved quantification when compared to manual segmentation methods.

VAMPIRE, the previously discussed nucleic analysis tool, is also capable of analysing cell membrane morphologies and produces measurements of membrane curvature using the same methodology. PCA analysis is used to determine a minimum set of measurands which describe cell shape features. K-means clustering is then used to classify cells into several shape modes, resulting in the identification of distinctive membrane morphologies (Phillip et al., 2021). Chen et al. also explored the classification of cell membrane morphologies using a machine learning-based methodology to identify individual bone marrow stromal cell (BMSC) phenotypes (Chen et al., 2016). Twenty-2 cell shape metrics were evaluated across three categories were quantified and correlation coefficients were calculated for each metric. Support vector machine (SVM) classification was then used to find the optimal classification boundary that separates data points in the multidimensional shape metric space. This method allowed for reduction in the number of shape metrics required to fully ascertain differing cell phenotypes, effectively identifying key critical quality attributes associated with cell membrane morphology. Furthermore, the impact of single-cell morphological heterogeneity was minimised by training the SVM classifier using averaged shape metrics taken from a randomly selected subset of cells known as ‘supercell’ averaging. Together, this analysis methodology identifies phenotypic changes in BMSCs in response to a range of microenvironmental cues. Despite this, VAMPIRE and SVM-based classification tools lack the ability to segment individual cells in densely populated cultures. These techniques present a potential opportunity to combine analysis methodologies from ACME to produce a more comprehensive tool for the in-depth analysis of cell membrane morphologies.

In addition to standalone image analysis software, several microscopes offer built-in software analysis tools capable of analysing cell membrane morphologies such as Imaris (Oxford Instruments), Incucyte software (Sartorius), and NIS-Elements (Nikon) (Gautier and Ginsberg, 2021; Roddy et al., 2016; Walton, Hofmeyr, and Van Der Horst, 2013). One advantage of built-in software is the ability to automatically consider cell imaging modalities and directly report image metadata alongside morphological outputs. Imaris, in particular, offers sophisticated analysis capabilities, such as the ability to segment closely associated cellular membranes, track cell lineage, and visualise cell surfaces. Whilst advantageous to Imaris users, a comparison study between instrument and standalone analysis tools would be required to determine the variability of output parameters. This evaluation would elucidate limitations of each analytical technique for complex cell analysis. Several studies have begun such efforts, with commercially available tools such as Imaris have been compared to manual tools such as ImageJ and independently developed research tools (Nguyen and Thompson-Peer, 2021; Gautier and Ginsberg, 2021; Rose et al., 2023).

Distillation of the necessary values for cell membrane characterisation is dependent on the organelle stains being co-stained alongside cell membranes. When combined with nuclei and actin measurements, cell area and edge intensity are valuable outputs when aiming to classify apoptotic or mitotic cells, due to the confirmational membrane changes that occur during each of those processes. Circularity, the measurement of how close a cell is to a true circle, and eccentricity, the measurement of membrane curvature deviations from circularity, are additional key outputs that provide information regarding cell membrane invaginations and extensions. These values reflect cellular response to external stimuli (López-Hernández, 2021; Lee et al., 2023). Measurements of curvature (rads/m) are traceable to S.I units and circularity, whilst dimensionless, can be measured with respect to reference materials with a known circularity, such as spherical beads of a similar size. It has also been shown by Phillip et al., that nuclei curvature measurements combined with cell classification offers a single concise measurement parameter that encompasses the morphological information described by circularity and eccentricity measurements, therefore distilling the number of potential CQAs related to cell membrane morphology (Phillip et al., 2021).

### 3.3 Actin image analysis methodologies and tools

Effective identification of individual cell actin cytoskeletons is typically reliant on first identifying cell nuclei which then act as seed points for the identification and segmentation of actin cytoskeletons. Propagation segmentation methods simply propagate out from the seed points and stop at intensity boundaries - this can be acceptable for cells which are generally uniform and circular. For cells which show irregular cytoskeletal morphologies, such as fibroblasts or smooth muscle cells, it is more appropriate to use Watershed or Laplacian of Gaussian (LoG) segmentation methods which use edge detection methods to segment images. Watershed algorithms determine cell boundaries using Sobel transformed images which compute a gradient intensity image function that seeks to emphasise edge detection. In contrast, LoG algorithms first smooth an image with a Gaussian filter to reduce noise. A Laplacian operator is then performed that computes the second spatial derivative of an image to enhance edge detection. Several analysis workflows have been developed that primarily aim to improve cell segmentation using watershed-based methods (Kucharski and Fabijańska, 2021; Wang, 2019).

Analysis of actin morphologies using CellProfiler is limited due to the simplistic measurement output values. Metrics such as actin area, edge and integrated intensity, eccentricity, and granularity provide generalised measurements of actin morphologies, however, more specific measurements of filament distribution, branching, and bundling are unable to be acquired. Nevertheless, the use of primary nuclei objects and option of further tertiary objects such as stained cell membranes allow for the quantification of object relationships which may produce information on cell bioactivity using known relationships. For example, a cell undergoing mitosis which show condensed, bright nuclei along with a rounded, contracted actin cytoskeleton.

**FIGURE 3:**
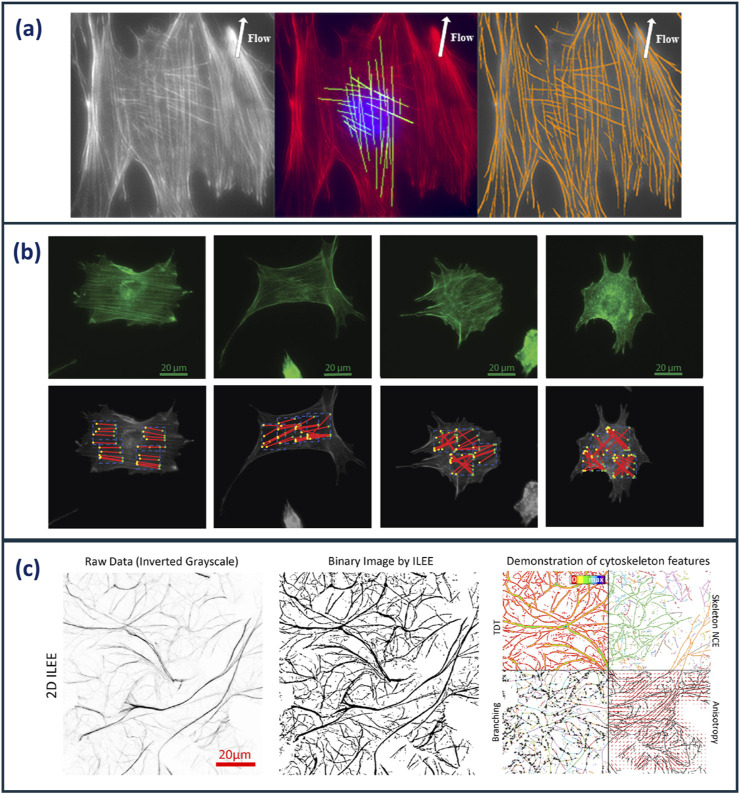
**(a)** *Images demonstrating the identification of individual, overlapping actin filaments in Osteoblast cells using the actin analysis framework developed by Alioscha-Perez et al. Cells were grown under fluid shear stress to observe alignment of actin filaments* (Alioscha-Perez et al., 2016) **(b)** *Example images cells analysed using IRAQ. The red lines highlight actin filament detection in cells exposed to increasing concentrations of latrunculin B (left to right)* (Liu, Mollaeian, and Ren, 2018). **(c)** *Images demonstrating the analysis workflow of ILEE. The raw image is binarised, then a range of 3D and 3D cytoskeletal features are computed* (Li et al., 2023)*. Images reproduced with permissions from respective publications.*

Several specialised analysis pipelines for the analysis of actin cytoskeletons have been developed that are capable of producing unique morphological indices. Alioscha-Perez et al. presented an actin analysis framework which focuses on individual filament morphological analysis (Alioscha-Perez et al., 2016). A three-step method was used to extract filament data which decomposed images into filament and texture components, then multi-scale line detector algorithm is applied to detect overlapping fibre segments from the basal and cap actin structures. Finally, filament segments were merged by overlapping segments with the same orientation connecting straight line segments according to their orientation difference up to a curvature threshold. This framework provides position, orientation, and length measurements of individual actin filaments. Figure 3a displays example images of the identification of individual, overlapping actin filaments using this analysis methodology.

Liu et al. (2018) developed an image-recognition based actin cytoskeleton quantification (IRAQ) methodology that uses edge, line, and brightness detection algorithms (Liu et al., 2018). Canny Edge and Sobel Edge detection algorithms are used to skeletonise actin images resulting in the output of three morphological parameters based on the orientation of the actin filaments: partial actin-cytoskeletal deviation (PAD), total actin-cytoskeletal deviation (TAD), and the average actin-cytoskeletal intensity (AAI). Figure 3b demonstrates fluorescent images of cells (top exposed to increasing concentrations of latrunculin B; a compound capable of perturbing the cytoskeleton). Below, actin filaments identified using IRAQ, demonstrates the ability of this tool to identify and quantify actin filament disruption. Weichsel et al. developed a similar actin analysis MATLAB-based code that quantifies stress fibres and the cortex at cell boundaries using a value known as Coherency. This value is defined through the structure tensor, which evaluates the local orientation in a small region of an image. This analysis tool particularly focuses on cell boundaries due to the limitations of optical microscopy in the identification of single actin fibres across an entire cell. This, in turn, allowed for high-throughput cell analysis resulting in robust statistical data (Weichsel et al., 2010).

Furthermore, Li et al. (2023) have recently developed an actin analysis algorithm employing the use of Implicit Laplacian of Enhanced Edge (ILEE) thresholding (Li et al., 2023). This sophisticated, unguided tool overcomes analysis limitations such as information loss from dimensional reduction, sample bias from manual user thresholding, and varying image performance from image quality. This algorithm is based on native brightness, first-order gradient derivative, and second-order Laplacian derivative characteristics of each input image. ILEE pipeline aims to automate the detection of cytoskeletons in an accurate unbiased manner. Figure 3c shows the outline of the ILEE workflow in which the raw image is binarised and used to compute a range of 2D and 3D cytoskeletal features. 12 cytoskeletal morphological indices are computed from this algorithm and categorised into five classes: density, bundling, connectivity, branching, and directionality. Whilst the complexity and comprehensiveness of this analysis tool is very effective at providing morphological information about actin cytoskeletons, it would be beneficial to distil down the number of output parameters and identify the CQAs required for cytoskeleton characterisation. Higaki et al. analysed the coefficient of variation of actin intensity as a means of describing the bundling of actin filaments. This was found to be comparable to skewness; the metric typically used to describe actin bundling. This demonstrates the ability of single metrics to describe multiple morphological features, reducing the number of indices required for characterisation (Higaki et al., 2020).

Overall, it is expected that a set of CQAs for cell actin morphology may consist of typical area and mean intensity data combined with specific indices acquired from the analysis tools described above. Filament length, density, and total number of nodes offer potential CQA values and are traceable to SI units of measurement (Daga et al., 2021). Values such as partial and total actin cytoskeletal deviation (PAD, TAD) may also be traced back to the SI unit of angular measurement, the radian, and can describe the directional organisation of the actin cytoskeleton with a single value. While Higaki et al. found that trend in bundling values largely agree with the coefficient of variation of actin intensity, to conclusively determine which actin output metrics are critical for the description of cell health, analysis of sample images set of known proliferative, apoptotic, and quiescent cells would be required (Higaki et al., 2020).

### 3.4 Mitochondria image analysis methodologies and tools

**FIGURE 4:**
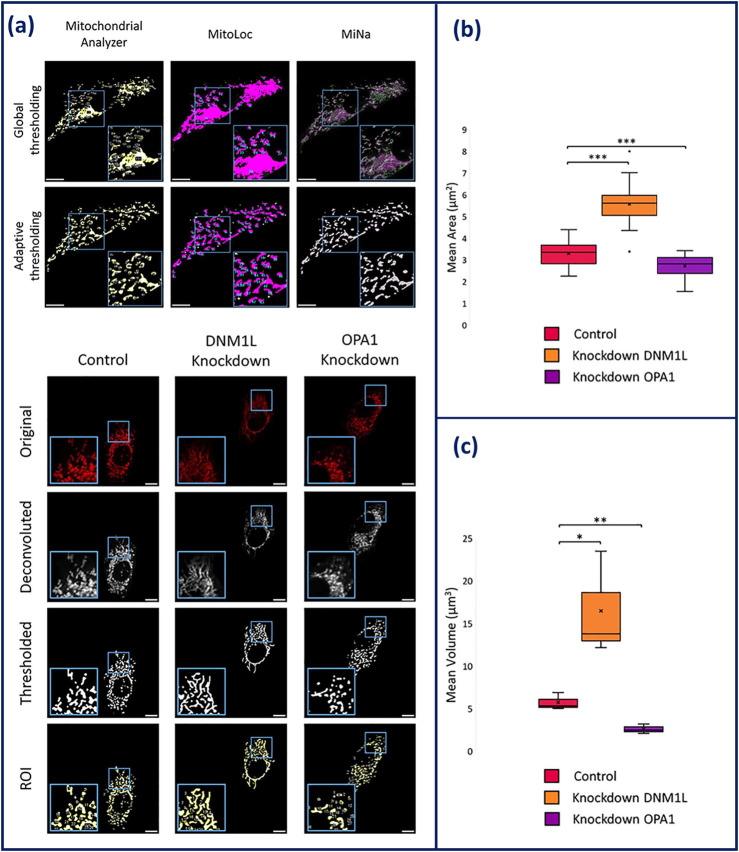
**(a)** Comparison of global and adaptive thresholding algorithms has on the effectiveness of mitochondrial network segregation within Mitochondrial Analyzer, MitoLoc, and MiNAa. **(b)** Overview of the imaging process of HeLa cells on Mitochondrial Analyzer. Elongation is shown in DNM1L knockdown cells and fragmentation is shown in OPA1 knockdown cells. **(c)** Statistical output of mitochondrial analyser showing mean area and volume of the mitochondria in knockdown conditions compared to control HeLa (Hemel et al., 2021). Figures have been adapted from publications with permission.

Improvements have been made in image processing algorithms and machine learning with several analysis pipelines being available for robust quantification of mitochondrial morphologies (Harwig et al., 2018). Current mitochondrial morphology analysis programmes have been developed as plugins for ImageJ and CellProfiler or are their own entity like MitoGraph and MitoER (Harwig et al., 2018). ImageJ has several different exclusive plugins including MiNa, MitoLoc, Mitochondrial Analyser and the Mitochondrial Morphology Macro (Valente et al., 2017; Hemel et al., 2021; Vowinckel et al., 2015; Chaudhry et al., 2020). The outputs of these plugins include number of fragments, mean area, and mean mitochondrial volume and in some instances, metrics describing mitochondrial branching. Branching is used as a method of describing the extent of mitochondrial networking, quantifying the amount of mitochondrial fission/fusion. Comparison of each of these analysis tools found that all were able to produce accurate outlines of the mitochondrial networks alongside measurement of the number of mitochondria. However, while measuring cellular mitochondrial clusters, MiNA, MitoLoc, and the Mitochondrial Morphology Macro were unable to separate mitochondrial structures. Due to integrated adaptive thresholding, Mitochondrial Analyzer was able to overcome the obstacle of incorrect segregation, recognising small structures within the mitochondrial network that are lost during filtering out the background signal thus, making it the most competent plug-in (Figure 4) (Hemel et al., 2021).

MitoGraph uses 3D images to measure individual mitochondrial structures as well as the volume of mitochondrial networking. Originally developed for quantifying mitochondrial volume in budding yeast, MitoGraph has more recently been validated to perform graph theory-based quantitative analysis of mitochondrial networks (Viana et al., 2015). This tool provides information about mitochondrial volume, total length, and degree of branching in the form of a connectivity score (Harwig et al., 2018). Although this software is validated against different cell lines, there are limitations to its success. It has been found that it fails to align 7.2% of nodes within the network and it struggles to segment the highly interconnected, often saturated perinuclear mitochondria. Despite its limitations, once optimised, it can batch analyse and can be a useful tool for high-throughput image analysis.

Along with MitoGraph, MitER is a newly developed open-source software which allows for the automated quantitative analysis of 3D mitochondrial morphology. The main outputs of the software include mitochondrial volume, surface area, and measurements related to inter-organelle contact focusing on contact with the mitochondria-endoplasmic reticulum (mito-ER). This software firstly utilises MitoGraph to process confocal images into single-cell 3D renderings. These renderings are processed in relation to the cell surface and input into MitER, where it is able produce quantifiable data on the mitochondria surface area, volume, spatial dimensions and number of Mito-ER contacts. For validation, quantifiable outputs were compared to MitoGraph outputs and were found to be agreeable with MitER benefiting from an automated workflow. Although this software has been optimised and validated for the measurement of mitochondrial morphology in yeast, the workflow has been adapted to mammalian cells. Mammalian cells pose the issue of irregular surfaces causing large rendering file sizes, ultimately causing slower analysis. To overcome this, a simpler mesh workflow is employed. With this modified workflow, the software was able to process mammalian images faster providing accurate morphological analysis (Kichuk et al., 2024).

Lastly, CellProfiler is an open-source software which allows users to identify key mitochondrial morphologies. As with actin, cell identification first requires nuclear staining, before segmentation and analysis of mitochondrial morphology. From this, CellProfiler can calculate the cytoplasmic area and determine mitochondrial networking and fragmentation. This program allows users to perform high-throughput analysis of mitochondrial images, but it has been shown to be less effective for acquiring data on extensive mitochondrial networking and is better suited in analysing fragmented mitochondria.

Overall, CQAs for mitochondrial morphological analysis may primarily include mean area, mean volume, and degree of branching. These outputs can provide data that is indicative of cell health and bioactivity based on mitochondrial morphology. For instance, this was shown when key genes related to mitochondrial morphology (OPA1 and DMN1L) were knocked-down, and morphological analysis tools were able to measure the impact of this knockdown. Upon knockdown of OPA1, fragmentation was observed and measured through the reduction in area and volume of the mitochondria. Whereas knockdown of DMN1L, presented an increase in area and volume, indicative of elongation demonstrated in Figure 4 (Hemel et al., 2021). By utilising mean volume and area of the mitochondria as a CQA, the measurement can be traced to the SI unit of meter, providing the prospective of standardisation measurement. This principle will be beneficial for future applications of mitochondrial morphological analysis, such as cancer detection and therapeutics (Chu et al., 2022).

## 4 Discussion and future Directions

This review has summarised the most notable analysis tools to characterise the nucleus, actin, mitochondria, and cell membrane structures. Key output metrics from each tool have been highlighted and assessed with reference to the identification of a concise set of measurable, quantifiable CQAs. Table 2 displays summary of each analysis tool, summarising their methodology, main output indices, and traceability to SI units. The review of cell analysis workflows revealed the initial challenge facing automated analysis tools is the effective segmentation of individual cell features. The extent of this challenge can be unique to each cell type and culture configuration but remains a crucial step in the analysis of individual cell profiles. Recent publications have shown that deep learning and AI-driven analysis tools are being developed to improve segmentation accuracy and reduce user analysis bias. However, there are considerable variations in the choice of deep learning algorithm and further alignment is required to encourage widespread adoption.

**TABLE 2:** Summary of the main analysis tools discussed throughout this review. Target organelle, analysis methodology, and main output indices are each described for each analysis tool.

Analysis Tool	Target	Analysis Methodology	Output Indices/S.I traceability
CellProfiler (Bray et al., 2014)	Nuclei, Cell Membrane, Actin, Mitochondria	Variable thresholding-based segmentation. Primary and secondary object identification enables multiple organelle analysis	Area/length measurements traceable to the meter. Intensity/circularity features require reference measurements for standardisation
Automated Mean Negative Curvature Analysis (Driscoll et al. 2012)	Nuclei	Automated extraction of nuclei boundaries followed by quantification of mean negative curvature using contour analysis	Mean negative curvature traceable to the meter and radian
Visually Aided Morpho-Phenotyping Image Recognition (Phillip et al., 2021)	Nuclei, Cell Membrane	Unsupervised classification of nuclei and membrane shapes using contour measurements to separate cells into shape nodes	Shape modes with shape factor, inertia, and aspect ratio. Shape measurements traceable to the meter and radian
SVM Supercell Classification (Chen et al. 2016)	Cell Membrane	Support vector machine (SVM) classification with supercell clustering used to identify the number of metrics required for cell classification	Minor axis length, solidity, and mean negative curvature all traceable to the meter and radian
Automated Cell Morphology Extractor (ACME) (Mosaliganti et al. 2012)	Cell Membrane	Cell boundary identification performed using Hessian-based filters to identify eigenvectors associated with boundaries. Well-suited for 3D tissues	Generic shape and intensity features traceable to the meter and radian
Robust Actin Filament Framework (Alioscha-Perez et al. 2016)	Actin	Three-step method using image decomposition into filament and texture components. Multi-scale line detection used to determine filament orientation and connectivity	Position, orientation, and length measurements of individual actin filaments. Orientation expressed in radians, and length in meters
Image-Recognition based Actin cytoskeleton Quantification (IRAQ) (Liu et al. 2018)	Actin	Canny and Sobel edge, line and brightness detection algorithms used to extract morphological information of filament orientation	Partial actin-cytoskeletal deviation (PAD) and total actin-cytoskeletal deviation (TAD) – both traceable to the radian. Average actin-cytoskeletal intensity (AAI)
Implicit Laplacian of Enhanced Edge (ILEE) algorithm (Li et al. 2023)	Actin	Tool uses native brightness, first-order gradient derivative, and second-order Laplacian derivative characteristics of input images to detect cytoskeletal features	12 morphological indices separated into 5 classes: density, bundling, connectivity, branching, and directionality. Traceability issues due to bespoke outputs
MitoGraph (Harwig et al. 2018)	Mitochondria	Automated image processing software dedicated to quantifying the volume of tubular mitochondrial networks	Mitochondrial total length, volume (traceable to the meter), and degree of branching (connectivity score)
MitoLoc (Vowinckel et al 2015)	Mitochondria	Automated 3D image processing to calculate and classify mitochondrial morphologies	Network morphology, fragmentation quantification (fragmentation index). Not directly traceable
MiNA (Valente et al. 2017)	Mitochondria	Analyses mitochondrial morphology using fluorescence images in 2D and 3D stacks. Estimates the volume and length of the mitochondria form a binarized copy of the image	Branch length, and mitochondrial volume – traceable to the meter
Mitochondrial Analyzer (Chaudhry et al. 2020)	Mitochondria	Analyses 2D, 3D and 3D time-series images of mitochondrial networks. It	Mitochondrial number, size, volume, and degree of branching (number of branches and length). Traceable to the meter

The review of nucleic analysis methodologies has revealed that a detailed profile of cell nuclei and DNA morphology can be obtained through the combined used of DNA and Lamin A/C labelling and subsequent analysis of nuclear size, intensity, and nuclear membrane curvature (Driscoll et al., 2012; Phillip et al., 2021). Similarly, quantification of cell membrane morphology can be achieved using similar metrics due to variations in cell area and cell membrane curvature. Curvature offers a traceable quantity of measurement with units of m^-1^ due to its definition as the inverse of the radius of curvature. Circular rainbow beads offer a means of providing reference curvature and fluorescence measurements which also serves to validate analysis methodologies. Furthermore, the m^-1^ unit can be applied to measurements using the same analysis methodologies as those used for nuclei. Tools capable of analysing the morphology of multiple organelles using the same methodologies increases the likelihood of widespread adoption and offer an attractive option for users without specialised knowledge.

Actin cytoskeleton and mitochondrial network analysis tools display the most sophisticated analysis methodologies due to the morphological complexity these cell features display. Significant conformational changes occur in both actin and mitochondria in response to cell bioprocesses and external stimuli, posing a morphological measurement challenge but also the ability to determine well-defined values relating to cell bioactivity. Outputs from ILEE and IRAQ actin analysis tools indicate that filament length, density, and total number of nodes are crucial to fully evaluating actin network morphology (Liu et al., 2018; Li et al., 2023). Outputs from MitoGraph and Mitochondrial Analyzer analysis tools indicate that mitochondrial volume and degree of branching/connectivity are key parameters which must be quantified to describe network morphology (Harwig et al., 2018; Hemel et al., 2021). The primary challenge with the quantification of these metrics is ensuring that the image detail and quality is sufficient for analysis. The requirement for high magnification and resolution of cell images presents time limitations when aiming to morphologically profile a representative proportion of a cell population–particularly if 3D Z-stacks are required. The feasibility of using each of these tools, or analysis methodologies derived from these workflows, could be tested by analysing a series of images taken at a range of magnifications and resolutions to determine the minimum image quality required.

It is evident that there are a range of analysis methodologies and tools used to quantify the morphology of individual cell features. Several analysis tools here have been developed to fit a broad range of analysis requirements and can therefore be implemented to multiple cell profiling assays (Laan et al., 2023; Phillip et al., 2021). Conversely, multiple analysis tools have been developed for specific purposes, often aiming to quantify the morphology of organelles undergoing specific processes (Liu et al., 2018; Driscoll et al., 2012). Whilst several metrics output from the tools discussed here overlap, there is still a significant number of user-defined values unique to each tool. This presents challenges in the identification of a minimal set of critical quality attributes, to describe the state of a population of cells through their morphologies. Furthermore, machine learning and AI-driven analysis presents a promising future for rapid and accurate morphological image and data analysis, highlighted by the current development ISO standards related to the use of AI in digital pathology (ISO/AWI 24051-2) and the increase in publications employing the use of machine learning for image analysis (Tourlomousis et al., 2019; Tang et al., 2024). However, from a metrological perspective, there remains uncertainties associated with the ‘black-box’ nature of many of these analysis tools that need to be addressed to improve current workstreams (Chandrasekaran et al., 2021; Piccinini et al., 2017; Chen et al., 2016; Phillip et al., 2021).

Identification of superfluous or coinciding output metrics could be performed by analysing reference image sets with each analysis tool. Principal component and linear discriminant analysis could then be used to identify linear combinations of variables which describe similar morphological features. Applying analysis of reference data sets to a range of cell types and treatment conditions could establish a robust set of CQAs which are able to describe a range of cellular bioprocesses while also potentially providing routes to perform quality assessment once they become established. This unmet requirement also presents a future opportunity for interlaboratory comparison studies, such as those carried out by CAWG (within CCQM). Such studies could look to compare output metric values from identical images disseminated to multiple laboratories, then progress to dissemination of biological samples for morphological analysis and ultimately determine how such CQAs can be included in measurement uncertainty calculations to underpin metrological traceability. This work would further previous CCQM pilot studies which have focused on cell enumeration through analysis of nuclei morphology. Such studies would inform the development of ISO guidelines which would advise the wider scientific community on best practices for developing and reporting on morphological cell analysis assays. Ultimately, the aim of such studies would be the development of a standard set of cell images which serve as a calibration tool to aid morphological image analysis data traceability and reproducibility. Researchers currently developing such assays should first aim to follow appropriate QUAREP instrument standardisation guidelines and further ensure that reported morphological data is traceable to standardised units or appropriate material standards.

## 5 Conclusion

This review has highlighted several morphological analysis tools used to quantify the morphology of key cell features commonly considered in cell profiling studies. The range of image acquisition modalities, image analysis algorithms, and output metrics has highlighted the variation and lack of standardisation across morphological profiling in academic and industrial sectors. It is evident that further alignment of methodologies is required to facilitate data robustness and reproducibility. Candidate CQAs for each cell feature have been highlighted and evaluated with respect to their prevalence, traceability, and practicality. Moving forwards, it is suggested that the testing of each analysis tool with a candidate reference image set would further investigate the efficacy of each analysis tool and aid in the identification of a minimal set of concise cell morphological measurement parameters. In general, progress in this field will be led by metrological institutions in close collaboration with both academic and industrial research institutions to ensure the needs of both communities are met. Reference methodologies and materials will aim to be developed using international case studies to determine method robustness and data comparability. The generation of a standard set of cell images, such as those used throughout digital pathology, could also serve as a calibration tool which would aid data traceability and reproducibility and facilitate the widespread adoption of morphological cell profiling.
